# Analysis of micro- and nanoplastics in wastewater treatment plants: key steps and environmental risk considerations

**DOI:** 10.1007/s10661-023-12030-x

**Published:** 2023-11-16

**Authors:** Simone Cavazzoli, Roberta Ferrentino, Costanza Scopetani, Mathilde Monperrus, Gianni Andreottola

**Affiliations:** 1 Department of Civil, Environmental and Mechanical Engineering (DICAM), University of Trento, Via Mesiano, 77 – 38123 Trento (TN), Italy; 2 Faculty of Biological and Environmental Sciences, Ecosystems and Environment Research Programme, University of Helsinki, Niemenkatu, 73 – 15140 Lahti, Finland; 3 Department of Chemistry ‘Ugo Schiff’ (DICUS), University of Florence, Via Della Lastruccia, 13 – 50019 Sesto Fiorentino (FI), Italy; 4 UMR 5254, Université de Pau et des Pays de l’Adour, E2S UPPA, CNRS, IPREM-MIRA, 64600 Anglet, France

**Keywords:** Organic-rich environmental sample, Extraction purification, Analytical method, Human ecosystem health, Organic–inorganic contaminant, Microorganism

## Abstract

The analysis of micro- and nanoplastics (MNPs) in the environment is a critical objective due to their ubiquitous presence in natural habitats, as well as their occurrence in various food, beverage, and organism matrices. MNPs pose significant concerns due to their direct toxicological effects and their potential to serve as carriers for hazardous organic/inorganic contaminants and pathogens, thereby posing risks to both human health and ecosystem integrity. Understanding the fate of MNPs within wastewater treatment plants (WWTPs) holds paramount importance, as these facilities can be significant sources of MNP emissions. Additionally, during wastewater purification processes, MNPs can accumulate contaminants and pathogens, potentially transferring them into receiving water bodies. Hence, establishing a robust analytical framework encompassing sampling, extraction, and instrumental analysis is indispensable for monitoring MNP pollution and assessing associated risks. This comprehensive review critically evaluates the strengths and limitations of commonly employed methods for studying MNPs in wastewater, sludge, and analogous environmental samples. Furthermore, this paper proposes potential solutions to address identified methodological shortcomings. Lastly, a dedicated section investigates the association of plastic particles with chemicals and pathogens, alongside the analytical techniques employed to study such interactions. The insights generated from this work can be valuable reference material for both the scientific research community and environmental monitoring and management authorities.

**Graphical Abstract:**

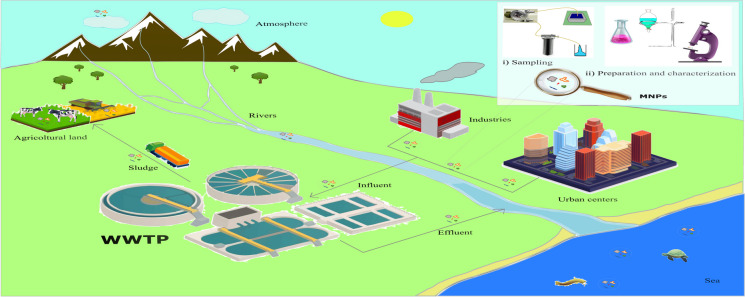

**Supplementary Information:**

The online version contains supplementary material available at 10.1007/s10661-023-12030-x.

## Introduction

In 2020, plastic production worldwide reached 267 million tons (PlasticsEurope, 2021). As countries recovered from the pandemic, these numbers only continued to rise (Li et al., 2023). Plastic durability and resistance to degradation are major contributors to the widespread plastic pollution we see today (Thompson et al., 2004). When plastic enters the environment, it is exposed to physical (e.g., photo-oxidation, mechanical wear), chemical (hydrolysis, oxidation), and biological (enzymatic catalysis) processes that break it down into smaller pieces, altering its material properties and forming MNPs (Zhang et al., 2021). These processes, known as weathering and aging, can also make the plastic more reactive and prone to further degradation (Uheida et al., 2021), flocculation, and sedimentation (Lee, 2012). Plastic polymers have unique properties, such as high durability, cost-effectiveness, and versatility, which make them suitable for a wide range of civil and industrial applications (Bacha et al., 2021). Plastics can be characterized by their size: mega- (> 1 m), macro- (< 1 m), meso- (< 2.5 cm), micro- (< 5 mm), and nano- (< 0.1 µm) plastics (GESAMP, 2019). The most common synthetic polymers on the market and in the environment are mainly derived from fossil resources, i.e., coal, petroleum, and natural gas. Among these, polyethylene (PE), polypropylene (PP), polystyrene (PS), polyvinyl chloride (PVC), and polyethylene terephthalate (PET) are representative plastic polymers (Geyer et al., 2017). MNPs found in the environment are generally divided into primary and secondary. The formers are intentionally produced to be micrometer-sized and include microbeads, microfibers, plastic pellets or nurdles, painters, and coatings. The latter, on the other hand, result from the degradation of larger plastic litter, which fragments over time due to the environmental processes mentioned above. When studying the dynamics of MNPs in the environment, numerous physicochemical and thermodynamic parameters need to be considered. For instance, the type of polymer influences the behavior and reactivity of the particles. The degree of lipophilicity of a polymer affects its ability to be bioaccumulated by an organism (Tourinho et al., 2019), as well as its propensity to adsorb contaminants from the surrounding environment (Rochman et al., 2013). Furthermore, among the crucial parameters in determining the fate of MNPs in the environment are their shape and size (Covernton et al., 2019).

Certain polymers, including PE, PP, and PS, are characterized by low buoyancy, allowing them to be transported over long distances (refer to Table 1). These polymers have even been detected in remote areas such as polar regions (Zhang & Chen, 2020), where they can accumulate in ice sheets and subsequently be released into surface waterways through melting processes (Bergmann et al., 2019). Rivers have been identified as significant contributors to plastic pollution in oceans, with a considerable portion of MNPs originating from inland areas (Browne et al., 2011). WWTPs play a critical role in intercepting and removing MNPs from wastewaters prior to their discharge into rivers and seas. For instance, Dris et al. (2015) conducted an assessment at the central Seine WWTP, which employs a conventional treatment process supplemented with biofilters in the biological stage. The authors estimated a removal efficiency for microplastics ranging from 83 to 95%. However, considering the substantial volumes of water treated by the Seine WWTP (240,000 m^3^/day), the microplastics released into the environment can still amount to millions of particles per day (Dris et al., 2015). MNPs tend to accumulate in sewage sludge (Bayo et al., 2016; Collivignarelli et al., 2021), commonly managed through landfilling, incineration, pyrolysis, or agricultural use as fertilizer (Rolsky et al., 2020). This practice may lead to the accumulation of MNPs in agricultural soils (Rodríguez Eugenio et al., 2018). The analysis of MNPs in WWTP samples is challenging, since the matrix to be treated consists of organic compounds, dissolved solids, pollutants, and microorganisms.

**Table 1:** Thermodynamical properties and general characteristics of the most common polymers found in the environment (data adapted from Bürkle GmbH, PlasticsEurope (2021), molinspiration.com, polymerdatabase.com, material-properties.org, makeitfrom.com)

*Polymer*	*Acronym*	*Natural color and state*	*Common applications*	*Density [g/cm*^*3*^*]*	*Water absorption (%)*	*Log P (monomers)*	*Max operating temperature*	*Min operating temperature*	*Lower heating value [MJ/kg]*	*Specific heat capacity [kJ/(kg·K)]*	*Tg [°C]*	*Tm [°C]*
*High-density polyethylene*	HDPE	Milky-white, flexible, and tough solid	Packaging, pipes and fittings, plastic bags, geomembranes and geotextiles, tanks and containers, playground equipment, agricultural applications	0.95	0.01	1.08	110	− 50	42.8–45.5	1.9	− 70	105–115
*Low-density polyethylene*	LDPE	Milky-white, translucent, flexible, and tough	Packaging, plastic films, squeeze bottles and containers, agricultural applications	0.92	0.01	1.08	95	− 50	42.8–45.5	2.3	− 100	120–130
*Polyamide*	PA	Ivory opaque, high mechanical, and chemical resistance	Textile and apparel, gears, toys, furniture, consumer goods, mechanical parts, and engineering components	1.13	1.30	2.15 (nylon 6,6)	90	0	Nd	1.6	~ 70	190–350 (nylon 6,6)
*Polycarbonate*	PC	Tough durable material, generally transparent to visible light	Electrical and electronic components, automotive industry, safety gears, construction materials	1.20	0.35	3.37 (Bisphenol A)	135	− 135	Nd	1.2–1.3	147	260–340
*Polyethylene terephthalate*	PETG	Amorphous (transparent) or semicrystalline (transparent/opaque)	Packaging material, medical applications, art, and crafts	1.78	0.70	1.80	70	5	Nd	~ 1	72	250
*Polypropylene*	PP	Rigid, opaque, yellow greyish	Packaging, consumer goods, automotive components, laboratory equipment, textiles	0.90	0.02	1.33	135	5	46.5	~ 1.9	100	130–171
*Polystyrene*	PS	transparent, rigid, and rather brittle	Packaging, insulation materials, disposable materials, building and construction	1.05	0.05	2.79	70	− 20	41.9	1.3	100	~ 240
*Polyvinylchloride*	PVC	White-yellowish, flexible to rigid	Construction material, vinyl flooring, electrical insulation, medical devices, packaging	1.35	0.06	1.22	70	− 30	Nd	1.4	82	100–260
*Polytetrafluoro* *ethylene*	PTFE	White solid, tough, and self-lubricated, flexible at high temperatures	Non-stick cookware, electrical insulation, seals and gaskets, bearings and bushings, industrial applications	2.25	< 0.01	1.45	270	− 270	Nd	~ 1	− 35	327

The presence of microplastics (MPs) in the environment has emerged as a critical concern due to mounting evidence linking them to potential adverse effects on both human health (Paul et al., 2020; Smith et al., 2018) and ecosystem integrity (Gaylarde et al., 2021; Kumar et al., 2021; Wang et al., 2021). Exposure of marine organisms to plastic contamination poses risks of microplastic bioaccumulation and subsequent chronic and/or acute toxicological impacts (Eerkes-Medrano et al., 2015). Furthermore, research has demonstrated the role of microplastics as carriers of hazardous substances, which can be released into the surrounding environment (Godoy et al., 2019; Santana-Viera et al., 2021). Environmental aging of plastic materials, influenced by exposure to prevailing conditions, can induce polymer structural changes and the formation of porous regions that act as sites for the exchange and adsorption of micropollutants (Vroom et al., 2017), subsequently facilitating their release (desorption) into the environment (Scopetani et al., 2022). Notably, the sorption and desorption phenomena of microplastics are significantly influenced by environmental variables such as pH, salinity, organic matter (OM), and particulate matter, as highlighted in Yu et al. (2019).

Studies have investigated the presence of MPs in various food and beverage products, revealing widespread contamination (Kosuth et al., 2018; Lwanga et al., 2017; Shruti et al., 2020). Therefore, investigating the presence of MNPs in wastewater treatment facilities is crucial to comprehend the role of WWTPs in seizing and releasing MNPs into the environment (Peng et al., 2017). Wastewater and sludge are known to be sources of organic and inorganic contaminants, pharmaceuticals, and pathogenic microorganisms (Rueda-Marquez et al., 2020). The transfer of these potentially hazardous agents into the environment through MNPs can pose an increased risk of environmental contamination. As of today, there is no specific legislation in Europe regarding micro- and nanoplastics in wastewater and environmental matrices. However, the issue has been addressed on several fronts in recent years. In 2018, the European Commission adopted the Plastic Strategy, to reduce plastic pollution and promote recycling. Within the Plastic Strategy, WWTPs are key systems to address decontamination of plastic litter. The European Chemicals Agency, on the other hand, oversees the risks associated with MNPs in commercial products and the environment, drafting in 2019 a guideline for their identification in such matrices. Regarding the protection of water resources and marine environments, the Water Framework Directive and the Marine Strategy Framework Directive require Member States to monitor and assess the presence and effects of potentially hazardous substances, including MNPs, and to take measures to reduce their environmental impact. Even at the international level, there is no specific regulation governing the presence of MNPs in wastewaters and in the environment. However, pioneer initiatives and guidelines aim to address the problem: In 2016, the United Nations Environment Programme published a report entitled “Marine Litter and Microplastics: Global Lessons and Research to Inspire Action and Guide Policy Change,” which highlights the environmental and health risks associated with the spread of microplastics in the environment, recommending actions to address the problem. The Organisation for Economic Cooperation and Development has published several reports on microplastics in the environment, including the “Guidance Document on the Fate and Behaviour of Microplastics in the Aquatic Environment.” The 2017 G20 also discussed the microplastic issue, adopting the “Marine Waste Action Plan,” which includes a commitment to reduce the discharge of plastic litter into the oceans. Although no stringent regulations exist to manage the global problem related to MNP contamination, the issue is relatively new, and regulatory frameworks are rapidly evolving.

This review examines the current state of MNP analysis in environmental matrices, primarily focusing on wastewater and sewage sludge, but also considering other environmental compartments such as marine waters, sediments, and soils. In the first part, we report the physicochemical characteristics that primarily determine the behavior of plastic polymers in the environment. The review then scopes in the analysis of MNPs within WWTPs, focusing on the sampling, extraction, and chemical analysis procedures. Emphasis is placed on critical points and procedural difficulties encountered during MNP analysis, as the development of a rigorous method is essential for assessing and limiting the distribution of such contaminants in the environment. In the last part of the review, we provide an overview of the organic and inorganic contaminants and microorganisms that can be found associated with MNPs, addressing the ecological risks that may arise from their release into the environment.

## Physicochemical properties of MNPs

The physicochemical characteristics and chemical resistances of commonly encountered plastic polymers in environmental matrices are comprehensively summarized in Tables 1 and S1 (Supplementary material). These tabulated values represent laboratory test results conducted on pristine microplastic raw materials, considering specific chemical-physical conditions such as temperature, pressure, construction characteristics, and chemical additives. Understanding the physicochemical properties of MNPs is essential for optimizing pre-treatment procedures and preserving their analyte characteristics, thereby preventing losses arising from degradation or transformation phenomena. Key factors influencing the chemical resistance of plastic polymers include the temperature of polymer-chemical compound contact, exposure duration, internal and external stress on the polymer, and the concentration of the chemical compound (Young & Lovell, 2011). The pH of liquid solutions in which MNPs are dispersed also plays a significant role in determining the chemical stability of plastic polymers. Moreover, certain physical and chemical properties of plastic polymers, such as density, transparency, water absorption, and n-octanol–water partition coefficient (*K*_ow_) of constituent molecules, can undergo changes due to structural alterations resulting from aging and weathering phenomena (Luo et al., 2020). Tabulated data in Table 1 highlights the varying operating temperatures for different plastic materials, with polytetrafluoroethylene (PTFE) exhibiting the broadest operating temperature range and the highest chemical resistance (Tab. S1). The same data suggest the capability of plastic polymers to withstand relatively high temperatures (70/90 °C) without undergoing degradation. However, two crucial considerations should be considered, namely the initial condition of the polymer (e.g., virgin, or aged and weathered plastic) and the particle size being processed. MNPs, due to their high surface-to-mass ratio, exhibit enhanced reactivity towards the surrounding environment (Singh & Sharma, 2008).

The physicochemical parameters mentioned above influence the fate of MNPs during wastewater treatment processes within WWTPs. Unfortunately, since these facilities are generally not specifically designed for the removal of MNPs, there are currently few technologies adopted to effectively capture MNPs during wastewater treatment. Given their resistance to biodegradation, physical methods such as fine-mesh filtration, membrane filtration, and centrifugation, as well as chemico-physical techniques like coagulation/flocculation/sedimentation, are primarily employed for MNP removal (Ali et al., 2021; Rout et al., 2022). Another potential approach for MNP treatment in wastewater is photocatalysis, a photochemical process that employs catalytic materials, organic, or inorganic, to accelerate photoreactions (Xu et al., 2021). From a commercial standpoint, photocatalysis-derived compounds resulting from MNP degradation, such as hydroxypropyl and butyraldehyde, may offer interesting prospects for applications in organic and pharmaceutical production (Uheida et al., 2021). Nonetheless, it is important to note that certain by-products of photocatalysis are currently under investigation due to their potentially hazardous nature (Tofa et al., 2019). In addition to physical and chemical methods, some studies have explored the use of microorganisms, such as actinomycetes, bacteria, and fungi to degrade various plastics, including PET, PE, PVC, and nylon (Amobonye et al., 2021; Chen et al., 2020; Scally et al., 2018; Shah & Alshehrei, 2017). Moreover, Xu and Bai (2022) demonstrated that MNP-contaminated sewage sludge could be treated through hydrothermal carbonization (HTC). This process immobilized plastic contaminants within the solid fraction of the HTC products (hydrochar) and led to their partial degradation during the treatment process.

### Quality assurance and quality control

To ensure the production of valid and representative data, the analysis of MNPs in environmental samples necessitates adherence to rigorous quality assurance and quality control (QA/QC) criteria. Given the widespread dispersion of plastic particles in the environment, the complete elimination of sample contamination during collection, extraction, and analysis procedures is challenging. Thus, utmost care must be taken during the sampling phase, employing non-polymeric materials, and employing isolation techniques to prevent atmospheric contamination. During sample preparation and analysis, minimizing the use of plastic materials, working within a laminar flow hood, and donning clean, cotton clothing are essential practices. To assess potential self-contamination, it is crucial to prepare and analyze experimental control blanks (negative controls, *n* ≥ 3) (Scopetani et al., 2020; Shruti & Kutralam-Muniasamy, 2023). It is crucial to prefilter the solutions and reagents used, as they may contain MNPs (Kutralam-Muniasamy et al., 2023). The glassware used during sample processing should also be liquid-rinsed with prefiltered solutions (Dehaut et al., 2019). Covering the samples with aluminum foil proves beneficial in preventing airborne plastic particle contamination, and mitigating potential photoreactions by maintaining the analytes in a dark environment. Prolonged exposure of MNPs to sunlight can indeed have a significant impact on their physicochemical characteristics (Du et al., 2021). Regarding organic-rich biological samples, such as (dehydrated) sludge, soil, and biota, they should be stored frozen at − 20 °C until MNPs are extracted (Hermsen et al., 2018).

To evaluate the impact of different sample purification steps on particle integrity, it is essential to conduct recovery tests (positive controls, *n* ≥ 3) using MNP standards. Several studies have reported changes, alterations, and degradation of MPs when subjected to aggressive reactive conditions and homogenization procedures. To date, there is a lack of comprehensive studies on the effects of sample preparation procedures on NPs. It is plausible that the experimental conditions that harm plastic microparticles may also affect plastic nanoparticles. This is especially true for chemical degradation, as nanoparticles are potentially more reactive due to their high surface area. However, this assertion requires further in-depth studies to be confirmed. Nuelle et al. (2014) employed various solvents (H_2_O_2_, NaOH, HCl) to remove biogenic material from marine beach samples. The authors observed damages, color changes, and size alterations in MPs exposed to hydrogen peroxide for seven days. Similarly, Li et al. (2020) investigated the purification of microplastics from sewage sludge, cattle manure, soil, sediment, and silicon dioxide samples using corrosive solutions (30% H_2_O_2_, Fenton’s reagents, and different concentrations of HNO_3_, HCl, and NaOH). PET, PA, and polymethyl methacrylate (PMMA) were found to be more susceptible to damage, while PS, PE, and PP exhibited little or no damage. Another study by Dehaut et al. (2016) successfully digested seafood samples with KOH and NaOH (60 °C, 24 h) for effective MP extraction, although the treatment caused shape and size changes in PET and polycarbonate (PC) particles. Additionally, Enders et al. (2017) observed modifications in Raman spectroscopic signals when MPs were exposed to oxidation solutions. Chemical treatments with an HNO_3_:HClO_4_ (4:1) acid mixture, saturated KOH solution, or KOH:NaClO 30% solution led to the formation of new Raman peaks, weakening of original peaks, and increased instrumental background noise for certain plastic polymers. Karami et al. (2017) employed similar chemicals (KOH, H_2_O_2_, HNO_3_, and HCl) at different temperatures and concentrations to digest fish tissues and recover MPs. The 10% KOH treatment at 40 °C yielded the highest organic tissue removal and MP recovery rate. However, the authors also observed degradation of PA-6, PA-6,6, and partial degradation of PET particles when incubated with hydrogen peroxide for 96 h. Fourier-transform infrared spectroscopy (FT-IR) images further revealed polymer degradation, with shifted analytical peaks and reduced peak intensity. PS exhibited a sharper peak at 998 cm^−1^ (corresponding to the ring breathing mode) after H_2_O_2_ treatment. In PP, the peak at 1116 cm^−1^ (representing C = C bond stretching) significantly decreased with HNO_3_ treatment. The same acid treatments on PET resulted in reduced peak intensities at 1610 cm^−1^ (C–C ring stretching) and 1722 cm^−1^ (C-O bond stretching). HDPE showed decreased intensities at 1288 cm^−1^ and 1432 cm^−1^ after HNO_3_ treatment, suggesting disorder in the lattice structure. For PVC, both HCl and HNO_3_ treatments reduced peak intensities at 627 cm^−1^ and 684 cm^−1^ (C–Cl bond stretching) and at 1420 cm^−1^ (C-H bending modes), potentially indicating conformational changes or denaturation of the polymer structure (Karami et al., 2017).

## Analysis of MNPs in WWTP samples

When analyzing WWTP samples, it is crucial to provide a comprehensive description of the plant’s characteristics. This includes details such as the location, average inflows and outflows, and the specific wastewater treatment technologies employed. These technologies may encompass processes such as coarse screening, grit removal, chemical treatment, primary sedimentation, biological treatment, and any tertiary treatment methods. Additionally, information on sludge management practices, including disposal methods and annual output, is valuable to include. It is also beneficial to report other data concerning the samples, such as chemical and biological oxygen demand, total organic carbon, total suspended solids (TSS), phosphorus, and nitrogen.

To delve further into the analysis of MNPs, it is necessary to distinguish between two categories: microplastics (0.1–5000 µm) and nanoplastics (NPs, 1–100 nm). This differentiation is essential due to the distinct extraction, identification, and quantification techniques required for each particle category. Currently, these particles cannot be analyzed together. Previous studies by Alimi et al. (2018), Nguyen et al. (2019), and Patil et al. (2022) have highlighted the necessity for separate analysis. The differentiation arises from the different properties characterizing these fractions, including chemical composition, structure, thermodynamics, electronic behavior, spectroscopic response, and electromagnetic properties, as discussed by Besseling et al. (2017).

### Microplastics

The National Oceanic and Atmospheric Administration (NOAA) protocol is one of the initial comprehensive methods for extracting MPs from various environmental matrices, primarily targeting water, beach, and seabed samples (Masura et al., 2015). This widely adopted protocol has been extensively utilized for analyzing MPs in organic-rich environmental samples like sewage, sludge, marine estuaries, sediments, and soils. Although the NOAA protocol offers relatively simple and universally applicable microplastic extraction steps, its size limitation (> 300 µm) and high temperatures (75–90 °C) used during analysis may not be suitable for fully investigating MP content in organic-rich samples. Additionally, the use of NaCl for densiometric separation needs reconsideration, as heavier polymers like PVC and PET (Table 1) may not float to the surface but settle to the bottom along with inorganic particles. Lastly, the identification and quantification techniques suggested by NOAA primarily rely on microscopy, which can introduce biases and lead to false identifications, including misinterpretations of plastic particles versus other particles and biomass (He et al., 2018; Song et al., 2015).

In addition to the NOAA-like method, various alternative procedures were proposed and implemented for isolating MPs from organic-rich samples such as freshwaters and sediments (Grbic et al., 2019; Imhof et al., 2012; Monteiro et al., 2022; Zhu, 2015). While these innovative methods address procedural gaps in MP analysis, their applicability may be limited to laboratory-scale analyses, posing challenges for large-scale implementation. On the other hand, certain methods have supplemented MP extraction procedures by suggesting additional purification steps to facilitate analysis. For instance, the use of digestive enzymes such as protease, lipase, cellulase, and chitinase has been explored in several studies (Löder et al., 2017; Mintenig et al., 2017a), and proteinase-K has shown promising results in removing organic matter from plankton-rich seawater samples (Cole et al., 2014a). Nevertheless, the wide array of MP extraction methodologies and variations in QA/QC criteria pose challenges in comparing results across studies. This chapter aims to explore the essential steps of a comprehensive protocol for microplastic analysis in wastewaters, sludge, and similar matrices, while assessing and discussing the strengths and limitations of various methodological approaches. Recommendations and procedural solutions will be provided to contribute to the development of a comprehensive protocol for analyzing microplastics in the environment.

#### Sampling

Wastewater treatment plant samples encompass influent and effluent water, intermediate treatment water samples, and sludge samples. To ensure representativeness, it is recommended to collect a minimum of 1 L for influent samples, with larger volumes suggested as the wastewater undergoes more treatment and purification. For instance, approximately 500 L of final effluent should be collected to obtain an adequate quantity of MPs (Koelmans et al., 2019). For influent sampling, a 24-h flow-weighted composite sample can be obtained using an auto-sampler commonly available in WWTPs for routine analysis. Representative volumes of effluent can be collected using a suitable 24-h sampling system that concentrates solids on a stainless-steel cartridge filter, similar to that employed by Mintenig et al., (2017). A flowmeter can also be used to monitor the water flow during the sampling period. The overall system allows the collection of a final sample representative of what enters the plant in 24 h. Designing the sampling system with a sequential series of filters featuring decreasing mesh sizes (e.g., 300–10–2 µm) allows for early distribution of solid particles during sampling, reducing the likelihood of individual filter clogging.

In addition to inflow and outflow, other sampling points within the WWTP are worth investigating. For instance, significant amounts of MPs have been observed in water extracted from sewage sludge during sludge dewatering processes using a centrifuge system (Salmi et al., 2021). Since such volumes of water are typically returned within the WWTP, any MPs present are likely to remain in the plant and potentially accumulate. Obtaining representative samples over a 24-h period is crucial for comprehensively assessing the water quality entering and leaving WWTPs. To ensure that representative samples are collected, it is useful to calculate the hydraulic retention times (HRT) of the wastewater within the plant, so that consistent matrices are sampled at the different sampling points. For instance, if the influent is sampled at time 0 and remains within the plant for 30 h, the effluent sampling should be conducted 30 h after. Accordingly, when sampling is performed after the primary settler and the HRT to that point is 2 h, sampling should occur 2 h after the influent sampling. However, this rationale may not be applicable to all matrices sampled within the WWTP. In the case of activated sludge (oxidation tanks, secondary biological treatment), the matrix exhibits homogeneity and approximate temporal constancy.

MP sampling can also occur via gravity capture on stacked stainless-steel sieves or filters, and manta or Neuston net (Ali et al., 2021; Sun et al., 2019). Samples can be grabbed or composite, and appropriate sampling material should be chosen to avoid sample contamination. For example, PVC and ethylene-propylene diene monomer (EPDM) are not desirable materials to be used in the sampling apparatus. The former is a polymer commonly found in WWTPs and in the environment (Rasmussen et al., 2021), while the latter could release polymeric microfragments, a phenomenon that should be further investigated to assess the extent, incident factors (e.g., aging and weathering of the hose material), and potential adverse effects on the environment. In contrast, PC is generally scarce in the environment (PlasticsEurope, 2021; UNEP, 2021), and is therefore more advisable for sampling, although ecotoxicological issues can be associated with the use of PC (Flint et al., 2012). Stainless-steel and silicone (polymerized siloxanes) pipes might be the best material for sampling, being chemically inert and durable and not suitable for microbial growth (Greenwood & Earnshaw, 2012). During sampling, precautions should be taken to avoid self-contamination, keep the sampling system’s pipelines clean, and conduct control tests to ensure the accuracy and reliability of the data.

When analyzing MPs in sludge (and soils) samples, the moisture and OM content in the initial samples obtained by drying at 105 °C and loss on ignition are usually reported (Ziajahromi & Leusch, 2022). Normally, 0.5–1 kg of sludge is collected as a bulk sample, possibly in stainless steel, glass, or other non-plastic containers, and kept covered, refrigerated, in the dark. Since sludge is known to accumulate microplastics, the 1 L sample can be divided into subsamples, of, e.g., 50 or 100 mL, and multiple replicated can be analyzed for statistical purposes (Mahon et al., 2017). This method is suggested, also considering the high content of organic material in sludge that must be removed during the sample preparation.

#### Wet sieving and filtering

When collecting aqueous samples, stainless-steel sieves are commonly employed for the separation of solid materials from the aqueous phase. It should be noted that the fractionation process at this point does not provide a precise definition of the granulometric classes of MPs, as they may be present in aggregates. However, it facilitates the subsequent extraction of MPs by reducing the operating volume. The NOAA method recommends a fraction size ranging from 5000 to 300 µm for analysis. Nevertheless, it is worth considering that the smallest MP fraction, i.e., particles smaller than 300 µm, may be the most prevalent in the environment (Hung et al., 2021). All plastic materials tend to gradually decompose and fragment into smaller particles once introduced into the environment; therefore, omitting the analysis of the smaller fraction of MPs would lead to an underestimation of their content in the samples. It is known that WWTPs can effectively remove larger plastic particles (Talvitie et al., 2017), while smaller fractions are more likely to bypass the purification stages, ultimately ending up in the seas and oceans. Talvitie et al., (2017) and Dyachenko et al. (2017) employed mesh sizes of 300, 100, and 20 µm and 5000, 1000, 355, and 125 µm, respectively, for sieving. In contrast, Ziajahromi et al. (2017) used a series of sieves with mesh sizes of 500, 190, 100, and 25 µm. It should be noted that sieving the initial sample with meshes smaller than 20 µm can be challenging, unless the solution to be filtered is exceptionally clean.

The sieving process entails passing liquid samples through a series of stacked sieves. The retained material is then transferred to a glass container using distilled water and a stainless-steel spatula. Non-polar organic solvents, such as n-hexane, can be used to transfer plastic microparticles between different containers (Dyachenko et al., 2017; Wolff et al., 2019). However, these solvents can react strongly with polymeric materials, especially with the smallest MP and NP particles, which possess a high specific surface area (Zhou et al., 2021). Alternatively, rinsing solutions that are not aggressive towards plastic polymers (refer to Supplementary table S1) could be employed, such as diluted citric acid or ascorbic acid solution, aqueous calcium carbonate or sodium bicarbonate solution, or an aqueous dextrin solution. The use of a diluted citric acid solution (e.g., 10%) can also enhance metal solubility and facilitate the Fenton reaction during the organic matter wet peroxidation step (Seol & Javandel, 2008), which may lose efficiency if the ferrous catalyst precipitates (Zhang et al., 2019). Nevertheless, it is important to note that the analysis of polymer-chemical resistances is an area that requires further investigation. The dissolution of MNPs could represent a complex pathway for transforming these contaminants into reusable products for chemical recovery or energy purposes (Chen et al., 2022).

In place of utilizing a sieve battery, MPs can be captured using a pressure sampling system, as detailed in “Sampling.” In alternative, centrifugation can be employed to separate the solid component, including MPs, from the liquid phase, as it was done by Monteiro et al. (2022) for organic-rich freshwater samples. This centrifugation-based approach was proposed as an alternative to sieving and demonstrated high efficiency in dewatering and MP recovery, without compromising the integrity of the MPs. However, it should be noted that the centrifugation method may face challenges when applied to large-scale systems with high organic matter loads, such as WWTPs. Other methods were proposed for collecting MNPs from environmental samples. For instance, Grbic et al. (2019) utilized hexadecyltrimethoxysilane-modified iron nanoparticles to bind dissolved MPs in freshwater and sediment samples, enabling their removal from the solution by exploiting the magnetic properties of the metal nanoparticles. Pressurized liquid extraction (PLE) has also been employed to extract different types of MP polymers from sediment, suspended matter, soil, and sewage sludge (Dierkes et al., 2019). However, it is important to note that PLE typically involves the use of non-environmentally friendly organic solvents, which can potentially damage the integrity of MPs.

Sieving or fractionating of sludge is a convenient method when the total solid concentration in the sample is low, particularly in the water line of a WWTP. It may be useful to report the moisture and OM content of the samples, which can be measured using a small aliquot of the initial sludge sample. The sieve mesh size should fall within the range of 0.1–5000 µm, and the retained material should be collected and stored with the assistance of purified water for further processing. Clusters of sludge, as can occur in soil, sediments, and compost, can trap plastic particles within them. To address this issue, salts like potassium metaphosphate (KO_3_P) can be used to disaggregate the sample and release any trapped particles. Another approach to separate MPs from clusters could involve the use of a mechanical grinder or an ultrasound bath treatment. However, it would be crucial to evaluate their potential contribution to plastic particle fragmentation.

#### Wet peroxidation

The Fenton reaction has been widely employed to eliminate OM from samples, which could otherwise interfere with the analysis of MP. This step is crucial for the effective separation and identification of MPs present in organic-rich matrices like wastewater and sludge. Notably, Tagg et al. (2015) demonstrated that wet peroxidation (WPO) using the Fenton reaction improves the quality of FT-IR analysis while having minimal impact on the properties of plastic particles. Fenton chemical oxidation, carried out at room temperature in the presence of a ferrous catalyst, is known for being environmentally friendly, cost-effective, and relatively easy to perform (Tagg et al., 2017). The efficiency of the reaction can be further optimized by utilizing Fenton-like processes, such as heterogeneous Fenton, photo-Fenton, electro-Fenton, cavitation-Fenton, and microwave-assisted Fenton processes. In some cases, these processes can be combined to enhance reaction efficiency (Wang et al., 2016; Zhang et al., 2019).

The Fenton reaction, as described in the NOAA method, involves using a mixture of 30% (v/v) H_2_O_2_ and a 0.05 M aqueous solution of FeSO_4_ in a 1:1 ratio. Prior to the reaction, most of the water in the sample is removed. The reaction is exothermic, with boiling occurring if the temperature exceeds 75 °C; therefore, temperature should be controlled by utilizing a cooling or ice bath. Stirring the solution at 75 °C for 30 min is the standard procedure for the Fenton reaction, with an additional 20 mL of 30% H_2_O_2_ added if necessary. In studies conducted by Tagg et al. (2017) and Hurley et al. (2018) to purify complex organic-rich matrices, the optimal concentration of Fenton’s reagent was found to be a 20 mg/mL FeSO_4_ solution with 30% (v/v) H_2_O_2_ in a 0.5:1 mL proportion. Tagg et al. (2017) worked at pH 5 and achieved good MP extraction results, while Hurley et al. (2018) worked at pH 3. The authors also evaluated potential MP losses resulting from the extraction procedures. However, it is important to note that both studies utilized relatively large pristine microplastics, which may be less susceptible to oxidative reactions compared to weathered and aged particles found in the environment. Considering that strong acidic conditions can potentially impact the integrity of plastic polymers (refer to Table S1, chemical resistance of polymers), it would be advisable to work at pH levels close to neutrality. Nevertheless, the optimal pH value for the Fenton reaction is 3 (Pignatello et al., 2006). As the pH of the WPO solution approaches 5, ferric and ferrous ions tend to precipitate as iron oxides and hydroxides, decreasing the availability of radical species such as •OH, thus lowering the oxidation efficiency of OM. Moreover, the precipitation of Fe_x_O_y_ and Fe(OH)_x_ results in the formation of red mud, which may entrap microplastic particles (which can act as nucleation centers) and make subsequent filtration steps of the oxidized sample challenging.

Various chemicals can be utilized to remove OM from sludge and analogous samples, such as soils and sediments. Among these are HCl, HNO_3_, NaClO, and strong alkaline solutions (NaOH, KOH). Hurley et al. (2018) investigated the effects of H_2_O_2_, Fenton reaction, and alkaline solutions (NaOH, KOH) on sludge and soil samples. They found that the Fenton reaction at pH 3 yielded the best OM removal results, with 86.9 ± 9.87% and 106 ± 13.8% removal rates in the sludge and soil samples, respectively, while preserving the integrity of MPs. In a study by Cole et al., (2014a, 2014b), different chemicals (HCl, NaOH, and proteinase-K) were tested at varying concentrations, incubation times, and temperatures to extract MPs from biota-rich seawater samples. The weakest digestive treatment was observed with HCl concentrations of 1 M and 2 M, whereas the use of proteinase-K resulted in the highest removal of OM (up to 97%). Furthermore, Herrera et al. (2018) examined different concentrations of extracting solutions (HCl, NaOH, KOH, NaOH + Sodium dodecyl sulfate (SDS), Fenton’s reagents, and ethanol) to evaluate the separation and recovery efficiencies of MPs in beached algae and plant samples. Results indicated a very high separation efficiency (up to 97%) with ethanol treatments, leveraging the density difference between the solution components. However, it is important to note that the fragments considered in this study were medium to large-size MPs (> 0.5 mm). The efficacy of the ethanol technique should be assessed with smaller MP particles to determine if an oxidative digestion step is still necessary to release MPs incorporated within the OM aggregates, as these particles may otherwise remain suspended along with the biogenic material. Mechanical techniques such as filtration and centrifugation have also been employed in conjunction with chemical treatments to enhance the separation of MPs from organic-rich samples. Monteiro et al. (2022) reported that centrifugation at 3500 rpm prior to chemical digestion (using KOH, Fenton’s reagents, and NaClO) and densiometric separation resulted in approximately 90% removal of OM. Regarding incoming municipal wastewater, it’s also worth noting that the samples to be treated are typically rich in toilet paper. For this reason, it may be necessary to use specific chemicals/enzymes to remove the paper, as its presence could cause interference during the chemical analysis of the sample.

Hurley et al. (2018) conducted experiments using H_2_O_2_, the Fenton reaction, and alkaline solutions (NaOH, KOH) on sludge and soil samples at different temperatures and concentrations. The most effective removal of OM was achieved with 30% H_2_O_2_ at 70 °C (up to 100% removal for both samples) and the Fenton reaction (up to 90% removal for both samples). However, the treatment of 30% H_2_O_2_ at 70 °C led to the destruction of PA-6,6 particles, and the 10 M NaOH treatment had an impact on PET and PC particles. In that study, the method of recovery was performed using pristine MP particles, and their size was in the order of 3 mm. Surfactants, such as SDS, may help purify OM-rich samples. Löder et al. (2017) used a sequential extraction method to separate MPs in plankton-rich seawater. The recovery of the method was tested on pristine MPs ranging from 150 to 500 µm in size. A first washing with the anionic surfactant SDS removed a significant portion (− 63.7 ± 4.3%) of the OM in the samples. Subsequent treatments with protease, cellulase, H_2_O_2_, chitinase, and ZnCl_2_ hypersaline solution further decreased the remaining OM. Overall, an average removal of 84.5 ± 3.3% of MPs was achieved by the end of the extraction process. Washing plastic particles with surfactants or purified water is indeed a useful step to remove any remaining oxidized OM from the surface of the plastic particles. An effective washing process might be essential for the instrumental analysis, as the presence of partially digested OM on the surface of MPs can lead to signal interference.

#### Density separation and filtration

By transferring the sample into a hypersaline solution, MPs having a lower density than this solution tend to float, while the heavier inorganic material settles. Saturated NaCl aqueous solution (density ~ 1.2 g/cm^3^) is commonly used being environmentally sustainable, affordable, and widely available. However, to recover heavy polymers, such as PVC and PET, denser hypersaline solutions are recommended. Among these, NaI and ZnCl_2_ can reach a density of 1.7/1.8 g/cm^3^, enough to separate polymers found in the environment. Li_2_WO_4_ solution (density ~ 1.6 g/cm^3^) has also been suggested for separating heavy polymer particles, but it is expensive and potentially carcinogenic (LMT Liquid, n.d.). To decrease operational costs and minimize waste production, the hypersaline solutions used in the density separation can be partially recovered through appropriate filtration (Rodrigues et al., 2020).

The density separation is typically performed using a separation funnel, where the post-WPO material is transferred, together with the hypersaline solution (Masura et al., 2015). Upon separation, the settled material is discarded, while the floating solids are captured on suitable filters (e.g., glass fiber filters, inorganic filter membranes) for solid–liquid separation. The retained sample can then be air-dried for 24 h in covered Petri dishes or transferred to a desiccator. The dried filters can be weighed to estimate the mass of microplastics within the environmental sample and stored until further analysis. WPO and density separation steps can be repeated to maximize the removal of organic and inorganic materials. Due to the small size of MP particles and their tendency to aggregate with organic and inorganic substances, it is advisable to remove the organic matter before density separation. This approach promotes the release of microplastics that may be incorporated into organic matter aggregates, thereby enhancing the effectiveness of subsequent density separation and overall extraction. A similar approach was chosen by Rolsky et al. (2020) to separate MPs from a municipal sewage sludge sample.

**Fig. 1:**
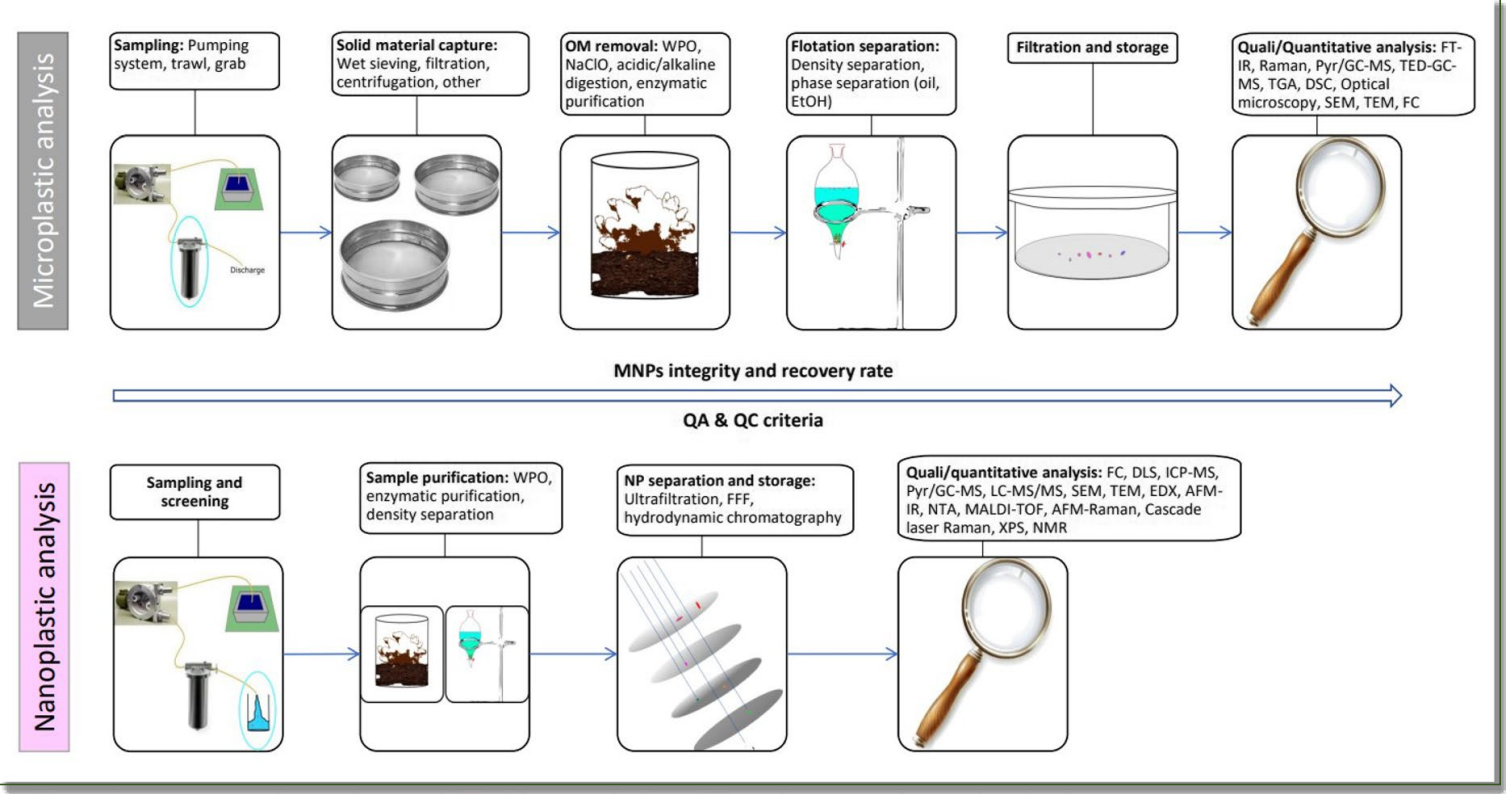
Rationalization of the protocol for the analysis of micro- and nanoplastics in environmental samples

Figure 1 reports a general outline of procedures for the extraction and analysis of MNPs in organic-rich samples (e.g., wastewaters and sludge).

#### Identification and quantification methods

Several analytical techniques are available for the identification, characterization, and quantification of MPs extracted from organic-rich samples. These techniques include the use of spectroscopic techniques such as FT-IR and Raman spectroscopy. Additionally, gas chromatography–mass spectrometry (GC–MS) analysis can be performed on pyrolyzed samples or samples extracted using thermal extraction desorption (Pyr-GC/MS, TED-GC–MS). Liquid chromatography methods can also be employed. Chemical analysis on purified samples is crucial to verify the synthetic origin of the analytes and expand the capabilities for detecting and quantifying MPs.

The use of a focal plane array (FPA) detector coupled with µFT-IR enables the identification and quantification of MPs directly on filters (Simon et al., 2018). However, FT-IR analysis can be time-consuming, so typically only a portion of the sample is analyzed. For example, the filter may be divided into quarters, and only one section is analyzed. Although statistical evaluation can assess the reliability of this approach, the resulting data may still lead to underestimation or overestimation of the actual MP content due to the typically non-homogeneous deposition of MPs on the filter. The detection limit of an FPA-equipped µFT-IR is approximately 10 µm, while particle-based µRaman spectroscopy can detect particles as small as 1 µm (Primpke et al., 2020). In Raman spectroscopy, the small size of MPs leads to weak Raman scattering signals, which are easily overshadowed by background noise, affecting sensitivity and detection limits. Therefore, strategies to enhance the signal-to-noise ratio and mitigate fluorescence interference are crucial. Moreover, the limited availability of comprehensive Raman and FT-IR signal databases and standardized reference materials for MPs hinders reliable identification and calibration, considering that degraded plastics give modified spectroscopic signals. Researchers are exploring the use of computer tools and artificial intelligence to improve MP analysis performance using spectroscopic techniques (Bianco et al., 2020; Lu et al., 2022). When reporting IR-based analytical results, it is important to include details about the FT-IR instrument used, any corrections for light reflectance or baseline shift, instrumental background analysis, spectra analysis method, and normalization, as highlighted by Andrade et al. (2020).

Thermoanalytical methods allow for the identification, and eventually quantification of MPs present in a sample by analyzing the semi-volatile products released during heating or by measuring the material and energy exchanged during the thermal treatment. Techniques such as Py-GC/MS, TED-GC–MS, differential scanning calorimetry, and thermal gravimetric analysis are examples of thermoanalytical methods used in MP analysis (Altmann et al., 2019; Becker et al., 2020; Duemichen et al., 2019; Fries et al., 2013; Hermabessiere et al., 2018; Majewsky et al., 2016). Although these methods are faster than spectroscopic techniques, they destroy MPs, preventing shape and size analysis. Therefore, the literature suggests combining multiple techniques for optimal results. The detection limits of thermal degradation methods coupled with GC–MS detection are very low, typically in the submicrogram range for each specific polymer (Schwaferts et al., 2019). However, the identification and differentiation of plastic polymers pose a critical challenge due to potential overlap with other organic and inorganic particles. For instance, Py-GC/MS relies on the identification of characteristic pyrolysis products for plastic differentiation. Alas, the varying chemical composition of plastics, copolymers and mixtures, and the presence of natural OM in the sample further complicate this task. Moreover, the sensitivity of Py-GC/MS may limit the detection and quantification of MPs, especially at very low concentrations. The small size (mass) of these particles adds to the challenge of achieving adequate signal intensity for accurate analysis. Standardized protocols for Py-GC/MS analysis of MPs are in early stages of development, necessitating method optimization encompassing pyrolysis conditions, chromatographic separation, and mass spectrometry parameters. Interpretation of complex pyrolysis mass spectra requires extensive knowledge and expertise. Developing comprehensive databases containing pyrolysis product profiles for various plastic types can facilitate MP identification in the future.

Flow cytometry (FC) can be applied to identify and quantify MPs in purified environmental samples. MPs analyzed with FC are previously stained with Nile Red or other suitable fluorescent markers, and the instrument analyzes scattered light and fluorescent signals, with a detection limit of 0.2 μm (Kaile et al., 2020). However, FC faces challenges with fluorescent marker aggregation, instrument calibration, and sample preparation accuracy (Adhikari et al., 2022). The viability of staining and resuspending filtered MPs for FC analysis is still being determined. Scanning electron microscopy (SEM) allows the study of structural and surface characteristics of MP particles and the identification of any degradation processes that have occurred on the particle surface, such as photocatalysis (Fu et al., 2020). The limit of detection (LOD) associated with the SEM technique is in the order of nanometers. In a recent study, SEM was combined with other analytical techniques to evaluate the impact of visible-light photocatalysis on pristine PP microplastics and the main photocatalysis products released into water (Uheida et al., 2021). Atomic force microscopy (AFM) is an innovative technique for studying submicrometric plastics. It can be coupled with spectroscopic methods to enable both physical characterization with high spatial resolution (50–100 nm) and chemical identification (Shim et al., 2017).

Physicochemical characterization of MPs should include the assessment of their shape, size, and color, as these structural characteristics can define the transport dynamics of MPs in the environment (Zhou et al., 2022). Additionally, the physical characteristics of plastic litter can provide insights into its source of emission, which can be associated with specific human activities. For instance, domestic washing machines can release a significant number of synthetic fibers into wastewater, which ultimately end up in WWTPs (Falco et al., 2019; Napper & Thompson, 2016). Similarly, the wear and tear of car tires can result in the release of a considerable amount of MPs, particularly in the form of black particles (Jan Kole et al., 2017). The quantification of MPs is typically expressed as the number of MPs per volume of wastewater or per (dry) mass of sludge. Alternatively, the concentration of MPs can be reported as the mass of MPs per volume (or dry mass) of the sample. This is the case of results obtained with Pyr-GC/MS analysis. For the analysis of WWTP samples, it is possible to estimate the amount (number, mass) of MPs entering and leaving the plant daily by considering the inflows, outflows, and the equivalent population treated by the specific WWTP. This information is valuable for assessing the MP removal capacity of the specific plant, in comparison to other facilities with different wastewater treatment technologies (e.g., conventional WWTPs vs tertiary treatments based on filtration or advanced oxidation processes). Moreover, emission data can be used to compare MP contamination levels in different geographic areas, such as mountains, plains, or delta regions.

### Nanoplastics

The potential entry of these MNPs into the trophic chain raises concerns regarding the associated harm to the organisms within it, ultimately leading to an elevated risk of ecosystem deterioration and biodiversity loss. In a groundbreaking study, Kashiwada (2006) investigated the effect of latex nanoparticles on *Oryzias latipes* (commonly known as “rice fish”) and suggested that these nanoparticles could traverse the blood–brain barrier and accumulate in the organism’s brain. Recently, Kopatz et al. (2023) corroborated these findings through their study on mouse models. The potential uptake of NPs by plants through the root system, subsequent systemic translocation, and accumulation within the plant body have also been demonstrated (Li et al., 2021). This possibility raises concerns regarding human and environmental health, particularly in relation to the presence of NPs in food items. Furthermore, Leslie et al. (2022) discovered polymeric plastic particles in plasma samples from public donors, providing evidence of systematic and ubiquitous human exposure to these contaminants. The contamination of MNPs has been associated with the degradation of soil quality and fertility (He et al., 2018; Hurley & Nizzetto, 2018). These particles have the potential to disrupt nitrogen biogeochemical cycles, alter the edaphic community, reduce enzyme functional diversity, and impact microbial biomass (Morgana et al., 2021). Such effects on the soil ecosystem are of great concern.

In WWTPs, the primary removal mechanism for NPs is through sludge absorption, as described by Rout et al. (2022). This process involves coagulation/flocculation phenomena occurring in settler and biological tanks, where the neutralization of repulsive charges among suspended particles leads to the settling and accumulation of NPs within the sludge. Recent experiments by Mitrano et al. (2019) showed that as TSS levels in biological sludge (secondary sludge) increase, NP concentrations also increase, as nanoparticles are probably captured within the sludge flocs. To enhance the effectiveness of these processes in WWTPs, coagulating agents such as Al_2_(SO_4_)_3_, FeCl_3_, or organic polymers can be added. These agents further destabilize the suspension and promote the separation of flocs by gravity, as explained by Bonomo (2008). An intriguing phenomenon involving dispersed nanoparticles is the formation of a protein corona around their surfaces. The composition and formation of this corona are influenced by various factors, including the surrounding environment and the integrity of micro- and nanoparticles, as discussed by Böhmert et al. (2020). The protein corona mainly comprises proteins, lipids, carbohydrates, ions, and water. In OM-rich aqueous matrices such as wastewaters, these constituents are abundant and diverse. The specific characteristics of the protein corona play a crucial role in determining the partitioning properties of micro- and nanoparticles between different phases and their assimilation into specific biological compartments, as highlighted by Wolfram et al. (2014).

#### Sampling and pretreatment

To date, limited research has been conducted on the presence of NPs in the environment, particularly within WWTPs (Ali et al., 2021). Several experimental studies have focused on the analysis of NPs in controlled conditions using simplified aqueous matrices that are spiked with virgin nanoparticle polymers. Notably, these samples may have significantly different characteristics compared to real environmental samples (Phuong, 2016). Analyzing plastic nanoparticles (0.001–0.1 µm) in organic-rich matrices is still fraught with numerous methodological and analytical challenges and limitations. However, the procedural steps for analyzing NPs in complex environmental samples, like those from WWTPs, remain largely the same as those required for analyzing MPs. This typically involves an initial sampling process followed by sample purification to obtain a material suitable for chemical analysis. Capturing NPs in wastewater samples can be performed using filters with suitable filtration porosity. For instance, commercially available filters can reach a pore size of 20 nm. These filtering membranes can be mounted on a dedicated in-line filtration apparatus to directly capture NPs during the sampling campaign. This sampling strategy was recently adopted by Xu et al. (2023). It is essential to sample enough NPs to perform the chemical analysis, and filtration is useful to concentrate NPs present in substantial volumes of water. Another sample preconcentration technique recently employed by Zhou et al. (2022) exploits the effects of an animal protein, the bovine serum albumin, which binds to NPs, forming a protein corona around them and making their capture easier. To separate NPs from inorganic matter in environmental samples, elutriation methods can be applied, similar to those used by Claessens et al. (2013) and Zhu (2015) for sediment samples. These methods can be combined with the purification steps described for MP analysis, such as WPO, to effectively remove undesired materials. It is important to note that neglecting the contribution of the smallest plastic particles, which may be the most abundant in the environment (Pérez-Guevara et al., 2022), can lead to a significant underestimation of the overall environmental plastic contamination.

#### Identification and quantification methods

Analytical methods commonly employed for nanomaterials can be adapted for the investigation of plastic nanoparticles (Bouwmeester et al., 2015). The zeta potential (*ζ*) and the *z*-average hydrodynamic diameter are typically measured to study NPs, as reported by Arenas et al. (2021). In their study, the authors examined the adsorption processes of polystyrene nanoparticles on granular activated carbon (GAC) using organic-rich lacustrine samples. They employed various analytical techniques including electrophoretic mobility and dynamic light scattering (DLS), along with SEM, to assess the surface adsorption rate, morphology, and aggregation state of NPs and GAC. Dispersed NPs can be separated and selected, based on their size, using ultrafiltration, flow field fractionation, and hydrodynamic chromatography. These pre-treatment steps are crucial in improving the resolving power of those analytical instruments that rely on light scattering measurements.

Flow cytometry can be used for the quantification and determination of submicrometric particles. As in the case of MP analysis, the fluorescent marker choice is crucial for accurate NP identification through FC. The phenomenon of NP autofluorescence can interfere with the analysis, while the presence of diverse polymers in organic-rich matrices can complicate the sample preparation (Caputo et al., 2021; Morgana et al., 2021). Nevertheless, FC provides high spatial resolution, enabling the detection of particles as small as 0.2 µm. Inductively coupled plasma mass spectrometry (ICP-MS) has been employed by Mitrano et al. (2019) to quantify Pd-coated polystyrene nanoparticles and indirectly assess their behavior within activated sludge. ICP-MS exhibits very low LODs, typically below µg/L. Scanning electron microscopy and transmission electron microscopy (TEM) are imaging techniques used to investigate the morphological characteristics of nanoparticles. SEM has a LOD of approximately 3 nm, while TEM offers even lower LODs (< 1 nm). Electron microscopy methods can be coupled with elemental analysis techniques, such as energy-dispersive X-ray spectrometry, to assess the elemental composition of particles (Caputo et al., 2021). Other analytical methods commonly used for the characterization of nanoparticles include nanoparticle tracking analysis (NTA) and DLS, as well as pyro-multi-angle laser light scattering (pyro-MALS) and atomic force microscopy-infrared (AFM-IR). The latter is particularly useful for investigating nanoscale surface features, such as chemical bonds and material properties, providing insights into hydrophobicity and conductivity (Luo et al., 2020). Asymmetric flow field fractionation (AF4) coupled with light scattering detectors, such as AF4-DLS or AF4-MALS, has shown promising results in the analysis of plastic nanoparticles within the range of 10 to 800 nm in diameter (Caputo et al., 2021). Additionally, AFM-Raman and cascade laser Raman techniques offer high spatial resolution for the detection and quantification of nanoscale particles (Zarfl, 2019). Matrix-assisted laser desorption/ionization time-of-flight mass spectrometry can also be used to characterize nanoplastics in complex organic-rich samples (Wu et al., 2020).

Pyr-GC/MS is a valuable method for the identification and quantification of NPs, and can simultaneously provide information on the organic contaminants and additives associated with them (Fries et al., 2013). However, the sample preparation is of paramount importance, since the presence of organic compounds, in addition to plastics, can interfere with measurements. Mintenig et al. (2018) proposed an analytical procedure for the analysis of both micro- and nanoplastics, involving cross-flow ultrafiltration to pre-concentrate analytes, followed by AF4-MALS and Pyr-GC/MS analysis. Similarly, a recent protocol was proposed for the quantification of MNPs in wastewaters, employing sequential microfiltration (1000–50–1 µm) and cross-flow ultrafiltration (~ 10 nm), hydrogen peroxide digestion, and density separation (for microplastics) or centrifugal ultrafiltration (for nanoplastics) (Xu et al., 2023). The authors collected 25, 50, and 100 L of primary, secondary, and tertiary settler effluents, respectively, using sampling buckets. MNPs were extracted and purified before Pyr-GC/MS analysis. Although methanol was used to remove any organic matter adhered to the plastic particles, and samples were dried at 90 °C, the authors reported no significant effects on the integrity of the MNPs (Xu et al., 2023). In another study, Li et al. (2021) employed Pyr-GC/MS to detect and quantify NPs absorbed by the root system of cucumber and systematically translocated within the plant. They followed a novel extraction protocol based on alkaline digestion, cellulose precipitation, and ultrasonic leaching. An additional batch of cucumber plants was exposed to a Pd core/PS shell dispersed nanoparticle solution, which were quantified using ICP-MS. NP particles can also be analyzed with liquid chromatography coupled with tandem mass spectrometry (LC-MS^2^). LC-MS^2^ is widely used in pharmacology, medicine, and environmental sciences, and can be applied to study volatile organic compounds. Similar to Pyr-GC/MS, LC-MS^2^ provides information on the total mass of polymers within the sample, particularly on the constituent monomers, as depolymerization is required prior to analysis (Adhikari et al., 2022). LC-MS^2^ is a promising technique, as it allows for the analysis of large sample quantities in a short time, estimation of molar masses of different polymer species, and quantitative analysis (Elert et al., 2017). X-ray photoelectron spectroscopy and nuclear magnetic resonance (NMR) have also been applied to NP analysis in environmental matrices. However, for these analytical techniques, the removal of unwanted organics from the sample is mandatory to minimize instrumental signal interferences (da Costa et al., 2016).

Caputo et al. (2021) tested nine different analytical techniques to measure the particle size distribution and mass concentration of polystyrene MNPs. They found that light diffraction failed to distinguish multiple populations of polydisperse particle samples, and NTA, FC, and AF4-MALS were not suitable for analyzing micrometer-sized particles. Moreover, TEM and FC were inadequate for quantifying the total particle mass. The authors proposed alternative techniques such as resistive tuneable pulse sensing and centrifugal liquid sedimentation for analyzing particles in the size range of 60 to 5 µm. Castelvetro et al. (2021) developed an innovative analytical procedure based on acidic/alkaline depolymerization followed by HPLC analysis to quantify the total mass of nylon-6, nylon-6,6, and PET MNPs in sewage sludge samples. Similarly, Corti et al. (2020) integrated a similar protocol with AFM-FT-IR to detect and quantify MNPs in lake sediments. The authors applied a multi-analytical approach, including 1H-NMR, which allowed them to accurately identify and quantify the polymers present in the samples.

## Risks of MNPs spread in the environment

### Exposure and hazards

Biological organisms in the environment can be exposed to contaminants through different routes, including ingestion, inhalation, and dermal absorption (Jiang et al., 2018; Rahman et al., 2021; Spalt et al., 2009). Exposure to airborne MNP can be significant, as 1 m^3^ of outdoor urban air can contain 1.5 MP particles, while 1 m^3^ of indoor air can contain up to 60. In the air, the majority of microplastics are in the form of microfibers, and the smaller fraction of these fibers (< 250 µm) can reach deep into the lungs (UNEP, 2021). Ingestion is considered an important route of exposure to MNPs, although numerous factors determine the overall exposure, including the physicochemical characteristics of the plastic particles, the type of exposed organism, and its living environment (Paul et al., 2020). MNPs have been found in fish, seafood, and many other foodstuffs such as vegetables, cereals, meat, honey, and salt, as well as in beverages including drinking water and beer (UNEP, 2021). Systemic ingestion of these particles can lead to oxidative stress, inflammatory reactions, and metabolic disorders in exposed individuals, and the extent of the damage was correlated with gender differences. In the human body, MNPs can bioaccumulate in the liver and kidneys and be transported through the lymphatic system (Prata et al., 2021; Wang et al., 2021). Because of their physicochemical properties, nanoplastics can be absorbed through skin tissue and penetrate the body (Bouwmeester et al., 2015).

**Fig. 2:**
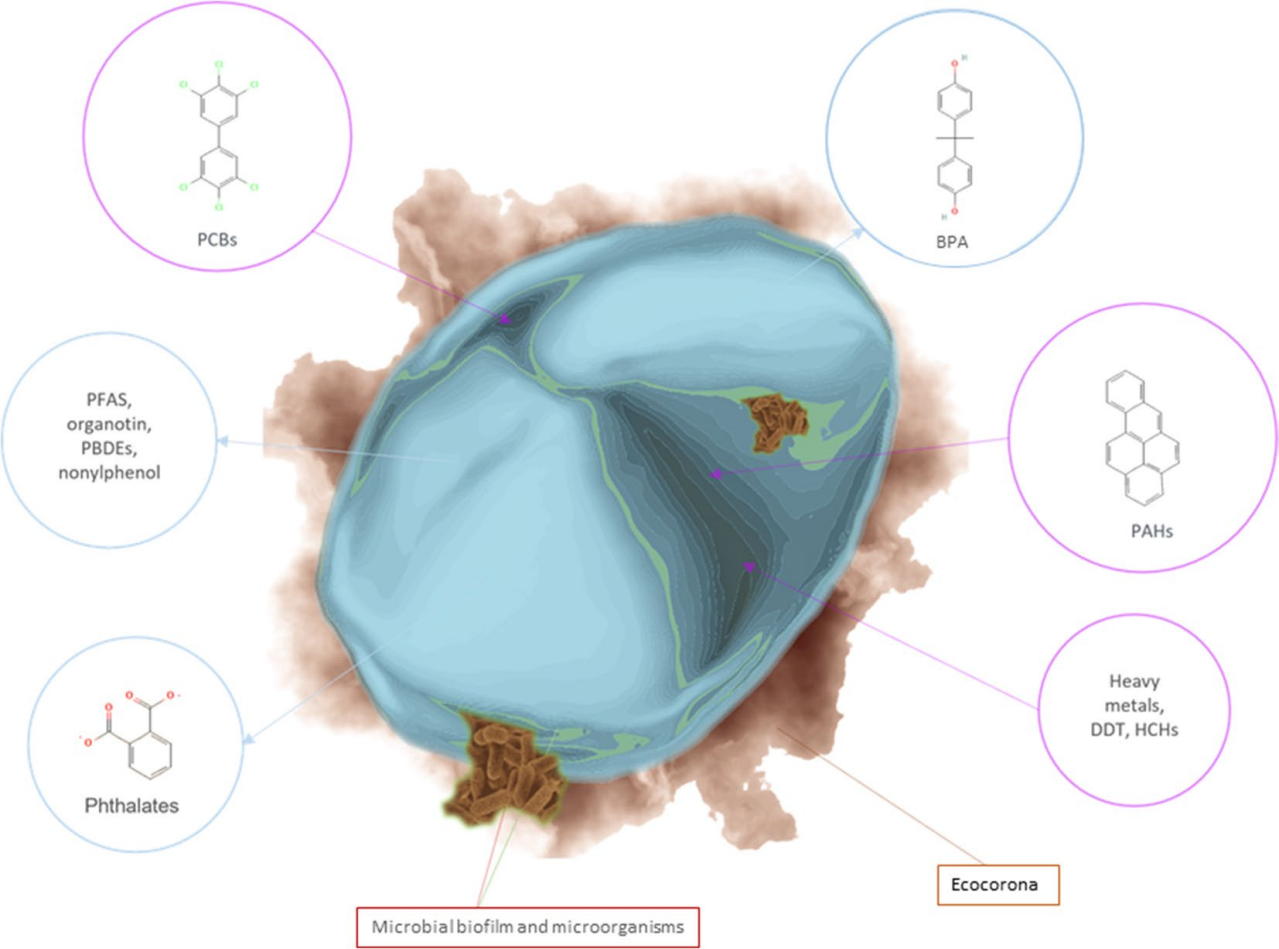
Schematic representation of the plastisphere complexity. Additives within the polymer structure can be released in the surrounding environment (light-blue arrows), while external contaminants can be adsorbed on the particle surface (purple arrows). Biogenic masses, e.g., bacterial cells and genes can be hosted on the particle

Plastic litter can easily be mistaken for food by animals. Acampora et al. (2016) examined the stomach contents of beached birds (*Fulmarus glacialis*) and found plastics in most individuals, which may have contributed to their death. Plankton-feeding animals such as whales and basking sharks can also ingest MNPs, and given the large volumes of water they filter they can be highly exposed (Koelmans et al., 2017). Meanwhile, predators can be exposed to plastics and associated contaminants if they bioaccumulate in their prey. Polystyrene nanoparticles were able to penetrate inside cells, suggesting a possible profound interaction between NPs and organisms (Rossi et al., 2014). A recent in vivo study using mouse models demonstrated how the characteristics of the ecocorona (Fig. 2) formed around MNPs can significantly influence the particle absorption capacity. Submicrometric particles (0.293 µm) surrounded by a cholesterol corona were observed to quickly cross the gastrointestinal barrier and penetrate the blood–brain barrier (BBB). However, this BBB penetration did not occur when the corona was composed of protein (Kopatz et al., 2023).

Ecotoxicological and epidemiological studies provide valuable information on the risks to human health caused by exposure to contaminants. MNP ingestion proved to have detrimental effects on organisms, including gastrointestinal blockage, damage to the digestive system, and disruption of the microbiome (Prata et al., 2021). Furthermore, MNPs can release associated organic and inorganic contaminants and additives under favorable conditions, such as those found in the gastrointestinal system. Assimilation of these contaminants by animals can lead to respiratory, cardiovascular, and metabolic diseases, neurodevelopmental disorders, and adverse effects on the reproductive system (Rahman et al., 2021). In vitro methods can be applied to estimate the bioaccessible fraction of contaminants released when a specific matrix interacts with digestive fluids. Through these methods, it is possible to assess the release of harmful substances from MNP in the gastric environment, where the presence of physiological surfactants such as bile can facilitate the process (Avio et al., 2015; López-Vázquez et al., 2022; Nathanail & McCaffrey, 2003).

Positive correlations were observed between the mass of ingested plastics and the amount of organic contaminants found in the adipose tissues of birds, confirming the contaminant transport pathway from the environment to plastics and then to organisms (Ryan et al., 1988). Experimental tests conducted on sediments demonstrated the distribution pattern of organic contaminants between MP particles, such as PE, PP, and PVC, and lugworms (*Arenicola marina*) (Teuten et al., 2007). In the case of small invertebrates, passive uptake can result in the overall accumulation of organic contaminants. This uptake likely occurs through dermal and/or post-digestive absorption, and the equilibrium between the concentration of the contaminant in the biota and the surrounding environment can be described by the partition constant *K*_d_ (Rice et al., 1993). The extent of contaminant bioaccumulation and trophic transfer depends not only on *K*_d_ but also on the rate of metabolic transformation of the compound within the organism (Wan et al., 2005). Organic contaminants adsorbed on MNP may be less bioaccessible, and their biodegradation can be inhibited. A study by Teuten et al. (2009) analyzing seabird preen gland oil samples found that PE can effectively preserve hydrophobic organic contaminants, such as polychlorinated biphenyls (PCBs), thus acting as a transporter to the organisms’ internal metabolic systems. Conversely, hydrophilic organic compounds are generally not bioaccumulative, as they are more readily metabolized by organisms (Chakrabarty, 1982).

Photolysis can lead to the formation of fractures and pores on the surface of plastic particles, promoting polymer degradation (Xu et al., 2021). Experiments have shown a significant increase in *K*_d_ when PE is subjected to prolonged irradiation, although polymer chain oxidations may decrease the hydrophobicity of the material and *K*_d_ values (Teuten et al., 2009). On the other hand, fouling and biofouling, along with contaminant sorption processes, can increase MNP sinking rates, contributing to the uneven distribution of these particles in aquatic systems. Lighter polymer particles, such as PE and PP, are more prone to settle on the seabed due to increased particle density or decreased hydrophobicity resulting from biofilm formation (Kowalski et al., 2016). Furthermore, extracellular polymeric substances produced by microorganisms and forming the biofilm make MNPs more attractive and easily ingestible by organisms, compared to pristine inert particles (Vroom et al., 2017).

### Hazardous chemicals from MNPs

Plastic polymers often contain organic and inorganic compounds added to enhance their properties. These additives can include phthalates, perfluoroalkyl substances, nonylphenols, organotins, bisphenol A (BPA), and flame retardants like polybrominated diphenyl ethers. These compounds are widespread in coastal seawaters and are suspected to be endocrine disruptors for wildlife and humans (Flint et al., 2012; Torres et al., 2021). On the other hand, organic contaminants such as dichlorodiphenyltrichloroethane and its metabolites, hexachlorocyclohexanes, and polycyclic aromatic hydrocarbons (PAHs) can be sorbed onto plastic particles from the surrounding environment. Plastic particles can also transport potentially harmful metals such as chromium, nickel, zinc, cadmium, and lead (Godoy et al., 2019). The adsorption of such metals onto plastic particles can be influenced by factors such as the biofilm formation on the particle surface and the degree of particle degradation (aging and weathering). These factors can enhance the reactivity of the particle, increasing the metal adsorption rate due to the presence of oxidized functional groups on the polymer chain (Yu et al., 2019). It is indeed suggested that particles exposed to the environment for longer periods may have a greater propensity to adsorb metals (Rochman et al., 2014).

PCBs can be sorbed on MNPs from the surrounding environment, and a high correlation was observed between the PCBs found on PP and PE particles and those absorbed by marine molluscs (Teuten et al., 2009). Due to their potential as adsorbers, plastic pellets made of PE, PP, PVC, and other polymers were proposed for monitoring potentially harmful hydrophobic chemicals in seas and oceans worldwide. Teuten et al. (2009) investigated the organic contaminants sorbed on marine-dispersed PE particles, finding that PCBs, dichlorodiphenyldichloroethylene, and PAHs were present on PE particles in all sampled areas. Polymeric organic sorbents have both crystalline and amorphous regions, with the amorphous regions being primarily responsible for sorption phenomena due to their characteristics related to the glass transition temperature (Reichenberg and Mayer, 2006). Diffusion of hydrophobic organic compounds through glassy polymers is slower than through rubbery materials, which behave more like viscous liquids (Teuten et al., 2009). Additionally, the diffusion rate is inversely proportional to the size of plastic particles. Glassy polymers are known to have nanovoids that exert strong adsorption forces, as described by Pignatello (1998). 

MNP floating on the surface of water environments are found in close proximity to those chemicals that naturally accumulate at the water surface, which can enhance the sorption process (Tourinho et al., 2019). The mobility of organic molecules within plastic particles depends on factors such as the pore size of the MNPs and the size of the molecules themselves, with smaller molecules being more mobile in larger pores (Reichenberg and Mayer, 2006). Plasticizers, which are commonly used additives in plastic materials, are not chemically bonded to the polymer chains and are susceptible to be released in the environment under favorable conditions (Gouin et al., 2011). Furthermore, the degradation of the plastic polymer structure can facilitate the migration of encapsulated molecules. The mobility of MNPs and the co-migration of different compounds are influenced by the thermodynamical properties of the organic molecules, and the environmental conditions such as temperature and pH. In the case of landfill leachates, rich in OM and with varying pH levels and microbial populations depending on the phase of the process (acidogenic or methanogenic), different organic contaminants are extracted from MNP over time. Hydrophilic organic additives, such as dimethyl phthalate and BPA, are released in the acidic leachate, while hydrophobic compounds like organotins and alkylphenols are more likely found in higher pH solutions, with lower ionic strength (Teuten et al., 2009). The release of endocrine-disrupting chemicals, such as BPA, in landfill leachate was associated with economic growth, urbanization, and industrialization, as measured by gross domestic product (Asakura et al., 2004).

Organic contaminants can be isolated from MNPs via different extraction techniques, including conventional liquid–solid extraction methods such as washing, maceration, shaking, soaking, and Soxhlet apparatus. Ultrasound-assisted extraction (UAE) and accelerated solvent extraction (ASE) are alternative methods that reduce the overall extraction time and lower solvent usage. Hexane and dichloromethane are commonly used to extract organic contaminants, and multiple extraction steps may be required to achieve optimal recovery of the analytes. After extraction, additional treatment steps such as drying, purification, and pre-concentration using solid-phase extraction may be necessary (Hong et al., 2017). These extraction procedures have demonstrated high sensitivity in terms of LOD and quantification. Traditional Soxhlet and liquid–solid extraction techniques were effective in separating organic contaminants adsorbed on MPs, but they can be time-consuming and require large amounts of toxic organic solvents (Hirai et al., 2011). UAE offers a more efficient extraction method with reduced solvent usage, while maintaining comparable effectiveness to liquid–solid extraction techniques (Llorca et al., 2014). On the other hand, ASE exploits the solubilization capacity of solvents at high temperatures and pressures, resulting in shorter extraction times and lower solvent volumes needed (Frias et al., 2010).

GC–MS is commonly employed to detect and quantify organic contaminants (Coquery et al., 2005). GC can be coupled with different types of detectors such as electron capture and flame ionization detectors. GC tandem triple quadrupole MS was used to analyze a wide range of organic contaminants associated with MPs (Camacho et al., 2019). Liquid chromatographic techniques including fluorescence detection, LC-MS^2^, and ultra-high-performance liquid chromatography tandem mass spectrometry can also be used to examine MP extracts containing dissolved organic contaminants (Llorca et al., 2014). Overall, these extraction and analysis methods provide researchers with valuable tools for studying the presence and characteristics of organic contaminants associated with MNPs.

### Microbes and antibiotic/metal-resistant bacteria and genes

As shown in Fig. 2, MNPs can host microorganisms, antibiotic-resistant bacteria, and genes, thus forming submicrometric environments known as plastisphere (Amaral-Zettler et al., 2020). The ecological importance of plastisphere communities is a critical research topic due to their potential influence on the behavior of MNPs in the environment and their interactions with organisms. Studies have demonstrated that plastics can serve as suitable microhabitats for bacterial cells such as *Escherichia coli* and *Vibrio* species (Galafassi et al., 2021; Harrison et al., 2014; McCormick et al., 2014). These pathogens can be transported to humans, as evidenced by cases where the presence of *E. coli* at bathing beaches exceeded regulatory limits, likely due to high concentrations of MPs carrying the bacterium (Rodrigues et al., 2019).

The microbial community within the plastisphere may differ from that of the surrounding environment, as it may derive from other sources. In WWTPs, disinfection treatments such as ozonation, UV-light, and chlorination can reduce both the abundance of pathogenic microorganisms and the amount of MPs in wastewaters (Kim et al., 2022). However, these treatments do not always succeed in completely removing pathogens, let alone plastic particles. In their research, Galafassi et al. (2021) investigated the bacterial community hosted by MPs in a WWTP effluent, as well as the presence of antimicrobial resistance genes, metal resistance genes, and class 1 integrons. They found that final ozonation treatments had no significant effect on the shape and size of most MPs, suggesting that such treatment is not able to remove these micropollutants. The antibiotic and metal resistance gene composition was significantly different in the plastisphere compared to the effluent, while the same composition did not change after ozonation. The bacterial community within the plastisphere was different from that in the wastewater, with the wastewater showing a higher abundance of potentially pathogenic bacteria. On the other hand, the plastisphere exhibited greater richness in bacterial genera, including *Chryseobacterium*, *Steroidobacter*, *Byssovorax*, *Nannocystis*, *Acidibacter*, *Legionella*, and *Tolumonas* (Galafassi et al., 2021). These findings align with other studies, confirming that MP particles serve as independent submicrometric habitats able to host diverse microbial niches (Di Cesare et al., 2020; Kirstein et al., 2016; Viršek et al., 2017). Plastic circulating in the environment can transfer not only microbes, but also more complex organisms, both marine and terrestrial, that can alter local ecological networks in habitats where they do not belong (Ali et al., 2021).

## Conclusions

To investigate the presence of micro- and nanoplastics in the environment, it is essential to follow a rigorous and reproducible methodology. Sampling must be designed to provide representative data of the target matrix. In the context of wastewater sampling, it is recommended to collect for a 24-h period, while periodic sampling campaigns are advisable to monitor contaminant level variations over time. The sample must then be purified to remove organic and inorganic substances. Among the various approaches available for organic substance removal, the Fenton reaction is efficient for high organic loads, as it swiftly purifies the sample while preserving the integrity of plastic particles. This rapidity can be advantageous for routine analyses conducted in laboratories within wastewater treatment plants. Inorganic substances can readily be removed through density separation, using appropriate hypersaline solutions to also recover the heavier polymer types. The micro- and nanoplastics extracted from the sample are then captured on a suitable filter, or dispersed in a solution, for subsequent analytical measurements. One of the aspects that make micro- and nanoplastics pollution of great scientific interest is the fact that they are capable of transporting and transferring chemicals and pathogens from one environment to another, increasing the risk of overall contamination and ecotoxicity. To mitigate emissions of micro- and nanoplastics into the environment, it is crucial to improve technologies and processes for their removal in wastewater treatment plants, as well as to adopt sustainable plastic waste management practices. A concerted effort is required to control the sources of these contaminants and establish comprehensive monitoring programs to track their presence in the environment.

### Supplementary Information

Below is the link to the electronic supplementary material.

Supplementary file1 (DOCX 44 KB)

## Data Availability

Not applicable. The submitted paper is a review, and the data can be found in the literature.
