# Supplementary material for: Analysis of micro- and nanoplastics in wastewater treatment plants: key steps and environmental risk considerations
Source: Environ Monit Assess. 2023 Nov 16;195(12):1483. doi: 10.1007/s10661-023-12030-x (PMC10654204; doi:10.1007/s10661-023-12030-x)
Supplement: Supplementary file 1 — Supplementary file1 (DOCX 44 KB) [file 10661_2023_12030_MOESM1_ESM.docx]

**Analysis of Micro- and Nanoplastics in Wastewater Treatment Plants: Key Steps and Environmental Risk Considerations**

Simone Cavazzoli^a*^, Roberta Ferrentino^a^, Costanza Scopetani^b,c^, Mathilde Monperrus^d^, Gianni Andreottola^a^

^a^ Department of Civil, Environmental and Mechanical Engineering (DICAM), University of Trento, Via Mesiano, 77 – 38123 Trento (TN), Italy

^b^ Faculty of Biological and Environmental Sciences, Ecosystems and Environment Research Programme, University of Helsinki, Niemenkatu, 73, - 15140 Lahti, Finland

^c^ Department of Chemistry ‘Ugo Schiff’ (DICUS), University of Florence, Via della Lastruccia, 13 - 50019 Sesto Fiorentino (FI), Italy

^d^ Université de Pau et des Pays de l’Adour, E2S UPPA, CNRS, IPREM-MIRA, UMR 5254, 64600 Anglet, France

*Corresponding author’s e-mail addresses : [simone.cavazzoli@unitn.it](mailto:simone.cavazzoli@unitn.it); [simonecavazzoli@gmail.com](mailto:simonecavazzoli@gmail.com)

Table S1 - Chemical resistance of common polymeric materials. Adopted from Wolff et al. (2019) and Bürkle GmbH, (2022). Two symbols: left = value at +20 °C; right = value at +50 °C. VV resistant, V practically resistant, X partially resistant, XX not resistant. One value = estimation; / = no data available; // = no general information available

| Chemicals | Concentration | Plastic polymer | | | | | | | | |
| --- | --- | --- | --- | --- | --- | --- | --- | --- | --- | --- |
|  |  | **HDPE** | **LDPE** | **PA** | **PC** | **PETG** | **PP** | **PS** | **PVC** | **PTFE** |
| Acid acetic (C_2_H4O_2_) | 100% | /-/ | /-/ | XX-XX | XX-XX | XX-XX | VV/X | /-/ | /-/ | VV-VV |
|  | 10% | VV-VV | VV-VV | XX-XX | VV-V | V-V | VV-VV | VV-VV | V-X | VV-VV |
| Acetone (C_3_H_6_O) |  | VV-VV | X-X | VV-/ | XX-XX | XX-XX | VV-X | XX-XX | XX-XX | VV |
| Ammonium hydroxide (NH_3_ + H_2_O) | 30% | VV-VV | VV-V | X | XX-XX | V-XX | VV-V | V-X | VV-V | VV-VV |
|  | 5% | VV-VV | VV-VV | V | X-XX | V | VV-VV | VV-X | VV-VV | VV-VV |
| Aniline (C_6_H_7_N) |  | VV-V | VV-X | X-XX | XX-XX | /-/ | V-X | XX-XX | XX-XX | VV-VV |
| Ascorbic acid (C_6_H_8_O_6_) | Aqueous | VV-VV | VV-VV | V | V | VV | VV-VV | /-/ | /-/ | VV-VV |
| Battery acid (38% H_2_SO_4_ in water) | 38% | VV-VV | VV-VV | XX-XX | VV-VV | XX | VV-VV | VV-VV | VV-X | VV-VV |
| Benzaldehyde (C_7_H_6_O) |  | VV-X | X-X | X-/ | XX-XX | XX-XX | VV-XX | XX-XX | XX-XX | VV-VV |
| Benzene (C_6_H_6_) |  | X-XX | X-XX | V-/ | XX-XX | XX-XX | X-XX | XX-XX | XX-XX | VV-VV |
| Benzoic acid (C_7_H_6_O_2_) | Saturated | VV-VV | VV-VV | X-XX | XX-XX | VV-/ | VV-X | V-V | VV-V | VV-VV |
| Benzyl alcohol (C_7_H_8_O) |  | X-XX | XX-XX | XX-XX | XX-XX | /-/ | XX-XX | XX-XX | V-X | VV-VV |
| Bromine (Br_2_) |  | XX-XX | XX-XX | XX-XX | XX-XX | XX-XX | XX-XX | XX-XX | V-XX | VV-X |
| Butyl acetate (C_6_H_12_O_2_) | 100% | V-V | V-X | VV-/ | XX-XX | X-/ | X-XX | XX-XX | XX-XX | VV-VV |
| Butyric acid (C_4_H_8_O_2_) |  | X-XX | XX-XX | X-X | XX-XX | /-/ | XX-XX | XX-XX | V-XX | VV-VV |
| Calcium bicarbonate (Ca(HCO_3_)_2_) | Saturated | VV-VV | VV-VV | VV-VV | VV | VV | VV-VV | /-/ | VV-/ | VV-VV |
| Calcium carbonate (CaCO_3_) | Saturated | VV-VV | VV-VV | VV-VV | VV-VV | VV-VV | VV-VV | VV-VV | VV-VV | VV-VV |
| Calcium phosphate (Ca_3_(PO_4_)_2_) | Aqueous | VV-VV | VV-VV | VV-VV | VV-VV | VV-VV | VV-VV | VV-VV | VV-VV | VV-VV |
| Calcium sulphate (CaSO_4_) | Saturated | VV-VV | VV-VV | VV-VV | VV-VV | VV-VV | VV-VV | VV-VV | VV-X | VV-VV |
| Camphor oil |  | XX-XX | XX-XX | V | X | /-/ | XX-XX | /-/ | XX-XX | V |
| Carbon dioxide (CO_2_) | Saturated | VV-X | VV-VV | VV-/ | VV-/ | VV-VV | VV-VV | VV-/ | VV-X | VV-VV |
| Carbon disulphide (CS_2_) |  | XX-XX | XX-XX | X-/ | XX-XX | /-/ | XX-XX | XX-XX | XX-XX | VV-/ |
| Carbon tetrachloride (CCl_4_) |  | XX-XX | XX-XX | XX-XX | XX-XX | V-XX | XX-XX | XX-XX | XX-XX | VV-VV |
| Cedar wood oil |  | X-XX | XX-XX | V | V-X | /-/ | XX-XX | XX-XX | X-XX | VV |
| Chlorine (Cl_2_) | Steam | XX-XX | XX-XX | XX-XX | XX-XX | XX-XX | XX-XX | XX-XX | XX-XX | VV-/ |
| Chloroform (CHCl_3_) | 100% | X-XX | XX-XX | X-XX | XX-XX | XX-XX | X-XX | XX-XX | XX-XX | VV-VV |
| Cinnamon oil |  | X-XX | XX-XX | V | V-X | /-/ | XX-XX | XX-XX | XX-XX | VV |
| Citric acid (C_6_H_8_O_7_) | 10% | VV-VV | VV-VV | VV-VV | VV-V | VV-X | VV-VV | VV-V | VV-X | VV-VV |
|  | Saturated | VV-VV | VV-VV | X-/ | V-/ | /-/ | VV-VV | VV-VV | VV-VV | VV-VV |
| Cresol (-mixtures, C_7_H_8_O) |  | X-XX | XX-XX | XX-XX | XX-XX | XX-XX | V-X | XX-XX | XX-XX | VV-VV |
| Dextrin ((C_6_H_10_O_5_)_n_ * H_2_O) | Aqueous | VV-VV | VV-VV | VV-VV | VV-VV | VV-VV | VV-VV | VV-VV | VV-VV | VV-VV |
| Dichloroethane (C_2_H_4_Cl_2_) |  | X-X | V-XX | X-/ | XX-XX | XX-XX | X-XX | XX-XX | XX-XX | VV-VV |
| Diesel fuel for heating |  | X-X | X-XX | VV-/ | X-X | VV-/ | VV-X | X-XX | VV-VV | VV-VV |
| Diethyl ether (C_2_H_5_)_2_O | Techn. pure | X-XX | XX-XX | VV-VV | XX-XX | VV-/ | XX-XX | XX-XX | XX-XX | VV-VV |
| Ethyl alcohol (C_2_H_6_O) | 96% | VV-/ | VV-X | VV-/ | VV-X | VV-VV | VV-VV | X-XX | VV-X | VV-VV |
| Ethyl acetate (C_4_H_8_O_2_) | 100% | VV-X | X-XX | VV-/ | XX-XX | XX-XX | VV-X | XX-XX | XX-XX | VV-VV |
| EDTA (C_10_H_16_N_2_O_8_) |  | VV-VV | VV-VV | V | V | V | VV-VV | /-/ | /-/ | VV-VV |
| Ferrous sulphate (FeSO_4_) | Saturated | VV-VV | VV-VV | V | VV-/ | /-/ | VV-VV | VV-/ | VV-VV | VV-VV |
| Fluorine (F_2_) |  | XX-XX | XX-XX | XX-XX | XX-XX | XX-XX | XX-XX | XX-XX | VV-V | VV-VV |
| Formic acid (CH_2_O_2_) | 98-100% | VV-VV | VV-VV | X-XX | X-XX | /-/ | VV-X | X-XX | X-XX | VV-VV |
| Gasoline (C_5_H_12_ – C_12_H_26_) |  | V-X | X-XX | VV-/ | X-X | V | X-XX | XX-XX | V-XX | VV-VV |
| Glycerol (C_3_H_8_O_3_) |  | VV-VV | VV-VV | VV-/ | X-X | VV-/ | VV-VV | VV-VV | VV-VV | VV-VV |
| Hexane (C_6_H_14_) |  | V-X | XX-XX | VV-/ | V | VV-/ | V-X | XX-XX | V-XX | VV-VV |
| Hydrogen chloride (HCl) | 35% | VV-VV | VV-VV | XX-XX | XX-XX | XX | VV-V | X-X | V-X | VV-VV |
| Hydrogen peroxide (H_2_O_2_) | 30% | VV-VV | VV-VV | XX-XX | VV-VV | VV-/ | VV-X | VV-V | VV-VV | VV-VV |
| Lactic acid (C_3_H_6_O_3_) | 3% | VV-VV | VV-V | X | VV-V | VV-/ | VV-V | V-V | V-X | VV-VV |
| Maleic acid (C_4_H_4_O_4_) | Aqueous | VV-VV | VV-VV | XX-XX | X | /-/ | VV-VV | /-/ | /-/ | VV-VV |
| Menthol (C_10_H_20_O) | Solid | VV-X | X-XX | X-/ | X-XX | /-/ | VV-X | XX-XX | /-/ | VV-VV |
| Methylene chloride (DCM) (CH_2_Cl_2_) |  | XX-XX | XX-XX | X-XX | XX-XX | XX-XX | X-XX | XX-XX | XX-XX | VV-VV |
| Nitric acid (HNO_3_) | 1-10% | VV-VV | VV-VV | XX-XX | VV-V | V | VV-VV | V-XX | VV-V | VV-VV |
|  | 70% | V-XX | X-XX | XX-XX | XX-XX | XX | XX-XX | XX-XX | X-XX | VV-VV |
| Nitrobenzene (C_6_H_5_NO_2_) |  | X-XX | XX-XX | XX-XX | XX-XX | VV-/ | V-XX | XX-XX | XX-XX | VV-VV |
| Nitrotoluene (C_7_H_7_NO_2_) | Techn. pure | VV-X | VV-X | XX-XX | XX-XX | VV-/ | VV-X | XX-XX | XX-XX | VV-VV |
| Octane (C_8_H_18_) |  | VV-VV | VV-VV | VV-/ | V-X | VV | VV-VV | XX-XX | X-XX | VV |
| Oleum (H_2_SO_4_ * SO_3_) | 10% SO_3_ | XX-XX | XX-XX | XX-XX | XX-XX | XX-XX | XX-XX | XX-XX | XX-XX | VV-/ |
| Oxalic acid (C_2_H_2_O_4_ * 2H_2_O) | Aqueous | VV-VV | VV-VV | XX-XX | V | V | VV-VV | /-/ | /-/ | VV-VV |
| Ozone (O_3_) |  | X-XX | X-XX | XX-XX | VV-V | /-/ | X-XX | V-V | VV-V | VV |
| Pectin | Aqueous | VV-VV | VV-VV | VV-VV | VV-VV | VV-VV | VV-VV | VV-VV | VV-VV | VV-VV |
| Phenol (C_6_H_6_O) | 10% | VV-VV | VV-VV | XX-XX | XX-XX | XX-XX | VV-VV | XX-XX | VV-X | VV-VV |
| Phosphoric acid (H_3_PO_4_) | 85% | VV-VV | VV-VV | XX-XX | VV-V | /-/ | VV-V | VV-V | VV-V | VV-VV |
| Potassium hydroxide (KOH) | 10% | VV-VV | VV-VV | VV-/ | XX-XX | XX-XX | /-/ | /-/ | /-/ | VV-VV |
|  | 30% | VV-VV | VV-VV | VV-X | XX-XX | XX-XX | VV-VV | VV-/ | VV-X | VV-VV |
| Potassium nitrate (KNO3) | 50% | VV-VV | VV-VV | VV-/ | VV-/ | VV | VV-VV | VV-VV | VV-VV | VV-VV |
| Potassium permanganate (KMnO_4_) | Aqueous | /-/ | /-/ | XX-XX | VV-/ | V | VV-VV | /-/ | /-/ | VV-VV |
| Propane (C_3_H_8_) | Gaseous | X-XX | XX-XX | VV-/ | X-XX | VV-/ | V-XX | XX-XX | VV-V | VV-VV |
| Pyridine (C_5_H_5_N) |  | VV-X | /-V | VV-/ | XX-XX | /-/ | X-X | XX-XX | XX-XX | VV-VV |
| Sea water |  | VV-VV | VV-VV | VV-/ | VV-VV | VV-VV | VV-VV | VV-VV | VV-X | VV-VV |
| Soap, liquid |  | VV-VV | VV-VV | VV-/ | V | V | VV-VV | /-/ | /-/ | VV-VV |
| Sodium bicarbonate (NaHCO_3_) | Aqueous | VV-VV | VV-VV | VV-/ | VV-/ | VV-/ | VV-VV | /-/ | /-/ | VV-VV |
| Sodium chloride (NaCl) | Aqueous | VV-VV | VV-VV | VV-VV | VV-VV | VV-VV | VV-VV | VV-VV | VV-VV | VV-VV |
| Sodium hydroxide (NaOH) | Concentrated | VV-VV | VV-VV | VV-X | XX-XX | XX-XX | VV-VV | /-/ | /-/ | VV-VV |
|  | 50% | VV-VV | VV-VV | VV-/ | XX-XX | XX-XX | VV-VV | V-V | VV-V | VV-VV |
| Sodium hypochlorite (NaClO) | Diluted | V-X | V-X | XX-XX | X | X-/ | V-X | VV-X | VV-X | VV-VV |
|  | 15% | V-X | V-X | XX-XX | V-X | X | V-X | VV-X | VV-VV | VV-VV |
| Sodium nitrate (NaNO_3_) | Saturated | VV-VV | VV-VV | VV-/ | VV-/ | V | VV-VV | VV-VV | VV-X | VV-VV |
| Steam (H_2_O) | Up to 150 °C | XX | XX | XX-XX | /-/ | / | X | / | / | VV |
| Styrene (C_8_H_8_) | 100% | XX-XX | X-XX | VV-VV | XX-XX | VV-VV | X-XX | /-/ | XX-XX | VV-VV |
| Sulphur dioxide (SO_2_) | Liquid | X-XX | XX-XX | X | X-XX | /-/ | XX-XX | XX-XX | X-XX | VV-VV |
| Sulphur trioxide (SO_3_) |  | XX-XX | XX-XX | XX-XX | XX | XX | XX-XX | /-/ | XX-XX | V |
| Sulphuric acid (H_2_SO_4_) | 1-6% (diluted H_2_SO_4_) | VV-VV | VV-VV | XX-XX | VV-VV | /-/ | VV-VV | VV-V | VV-V | VV-VV |
|  | 60% | VV-X | VV-X | XX-XX | X-X | XX | VV-X | V-XX | VV-V | VV-VV |
|  | 98% (concentrated, fuming) | XX-XX | XX-XX | XX-XX | XX-XX | XX-XX | XX-XX | XX-XX | XX-XX | VV-/ |
| Tetrabromoethane (C_2_H_2_Br_4_) | 100% | XX-XX | X-XX | X | XX-XX | XX | X-XX | XX-XX | XX-XX | VV |
| Tetrachloroethylene (PCE) (C_2_Cl_4_) |  | XX-XX | XX-XX | XX-XX | XX-XX | X-XX | XX-XX | XX-XX | XX-XX | VV-VV |
| Tetrahydrofuran (THF) (C_4_H_8_O) |  | X-XX | XX-XX | VV-/ | XX-XX | VV-/ | X-XX | XX-XX | XX-XX | VV-/ |
| Tribromomethane (CHBr_3_) |  | XX-XX | XX-XX | XX-XX | XX-XX | XX-XX | XX-XX | XX-XX | XX-XX | VV |
| Urea (CH_4_N_2_O) | Aqueous | VV-VV | VV-VV | VV-/ | VV-VV | VV-/ | VV-VV | /-/ | /-/ | VV-VV |
| Vinylidene chloride (C_2_H_2_Cl_2_) |  | XX-XX | XX-XX | V | XX-XX | XX-XX | XX-XX | XX-XX | XX-XX | VV |
| Xylene (C_8_H_10_) |  | X-XX | X-XX | VV-/ | XX-XX | /-/ | XX-XX | XX-XX | XX-XX | VV-/ |
| Zinc chloride (ZnCl_2_) | Aqueous | VV-VV | VV-VV | X-XX | V | /-/ | VV-VV | /-/ | /-/ | VV-VV |
| Zinc oxide (ZnO) | Solid | VV-VV | VV-VV | VV-VV | VV-VV | VV-VV | VV-VV | VV-VV | VV-VV | VV-VV |
